# Developing cloud applications using the e-Science Central platform

**DOI:** 10.1098/rsta.2012.0085

**Published:** 2013-01-28

**Authors:** Hugo Hiden, Simon Woodman, Paul Watson, Jacek Cala

**Affiliations:** School of Computing Science, Newcastle University, Newcastle upon Tyne NE1 7RU, UK

**Keywords:** e-Science, cloud computing, workflow

## Abstract

This paper describes the e-Science Central (e-SC) cloud data processing system and its application to a number of e-Science projects. e-SC provides both software as a service (SaaS) and platform as a service for scientific data management, analysis and collaboration. It is a portable system and can be deployed on both private (e.g. Eucalyptus) and public clouds (Amazon AWS and Microsoft Windows Azure). The SaaS application allows scientists to upload data, edit and run workflows and share results in the cloud, using only a Web browser. It is underpinned by a scalable cloud platform consisting of a set of components designed to support the needs of scientists. The platform is exposed to developers so that they can easily upload their own analysis services into the system and make these available to other users. A representational state transfer-based application programming interface (API) is also provided so that external applications can leverage the platform's functionality, making it easier to build scalable, secure cloud-based applications. This paper describes the design of e-SC, its API and its use in three different case studies: spectral data visualization, medical data capture and analysis, and chemical property prediction.

## 1. Introduction

Cloud computing has the potential to revolutionize e-Science by giving scientists the computational resources they need, when they need them. On their own however, clouds do not make it easier to design, implement and maintain the scalable, secure and dependable applications needed to support scientists. The problems can be seen when the various levels of cloud computing offerings currently available are considered [1].

Infrastructure as a service (IaaS): this is typical of many cloud offerings—for example, Amazon EC2 [2]. Using IaaS, developers can dynamically provision compute and storage resources, and they typically have control over the whole software stack including the operating system. The drawback is that for the majority of potential scientific users, access to raw hardware is of little use as they lack the skills and resources needed to design, develop and maintain the robust, scalable applications they require.

Platform as a service (PaaS): provides a higher level of abstraction than IaaS, as developers are provided with a platform containing services that can be used to build applications. For example, force.com [3] provides a variety of hosted services that can be used to develop customer relationship management-related applications in the cloud. The drawback is that, for commercial reasons, current platforms focus on services required for business applications, rather than those needed for scientific data storage and analysis (in §2, we give our view of the platform services that are needed to support scientific applications, based on our experiences in working with a wide range of scientists over the past 10 years).

Software as a service (SaaS): makes packaged applications available to users through the Web. Examples include Google Docs and salesforce.com [4]. Again, the problem is that the applications provided to date have focused on the large, commercial markets such as e-mail and document management. Some of these functions may be useful to scientists (for instance, Google Charts), but they do not meet the full range of needs of scientists.

As a result of these limitations, we have concluded that there will be relatively few science research groups with the skills and resources required to build scalable, secure and dependable science applications on the existing cloud offerings. The danger, therefore, is that the potential of the cloud to revolutionize e-Science will not be fully realized.

To address this, we have designed e-Science Central (e-SC), a cloud-based science PaaS that allows scientists to store, analyse and share data in the cloud. e-SC has now been in constant use for over four years, with over 300 users. In this time, over two million workflow executions have been enacted on the system.

This paper's main contributions are to describe:

— the platform's main cloud services, which are: data storage, service execution, security, workflow enactment and provenance. These have been designed to be independent of any specific cloud infrastructure: e-SC can run on both private clouds (e.g. Eucalyptus [5]) and public clouds (e.g. Amazon EC2 [2] and Microsoft Windows Azure [6]);
— the application programming interface (API) provided to allow users to develop and upload new services to run on the cloud platform and for external applications to access data, code and workflows deployed within e-SC; and
— a set of case studies in which the e-SC API was used to develop external applications; these are drawn from a range of different applications areas: medicine, materials science and cancer research.

This paper is structured as follows: §2 describes the overall functionality of e-SC as exposed through its Web interface; §3*a*,*b* then describe key platform services and the API exposed by the platform; §4 demonstrates the use of this API via a number of case studies; and finally we draw conclusions in §5.

## 2. e-Science Central overview

This section describes the overall functionality of e-SC as exposed through its science SaaS, Web 2.0 interface (www.esciencecentral.co.uk). e-SC was created in 2008 as the result of our work with a wide range of scientists over many years. This work had identified four key requirements that users required from an e-Science platform: the need to store data, analyse it, automate the analysis and share data in a controlled way. The e-SC platform created as a result of this experience allows users to do this entirely through a Web browser. We chose to offer this Web-only approach after investigating reasons for the lack of uptake of some existing e-Science tools for university research, and industry research and development—it became clear that many groups do not have the skills or resources needed to deploy and maintain applications, while many organizations actually prevent users from deploying their own software. Further, the browser-only approach suits the new way of working for many scientists, who wish to do their work wherever they are, on a mixture of systems ranging from mobile devices to laptops, tablets and desktop PCs, both at home and at work. Since its initial development, the scope of e-SC has evolved from being a simple data storage and sharing facility to the provision of a complete system that also allows data analysis and provenance capture.

**Figure 1.**
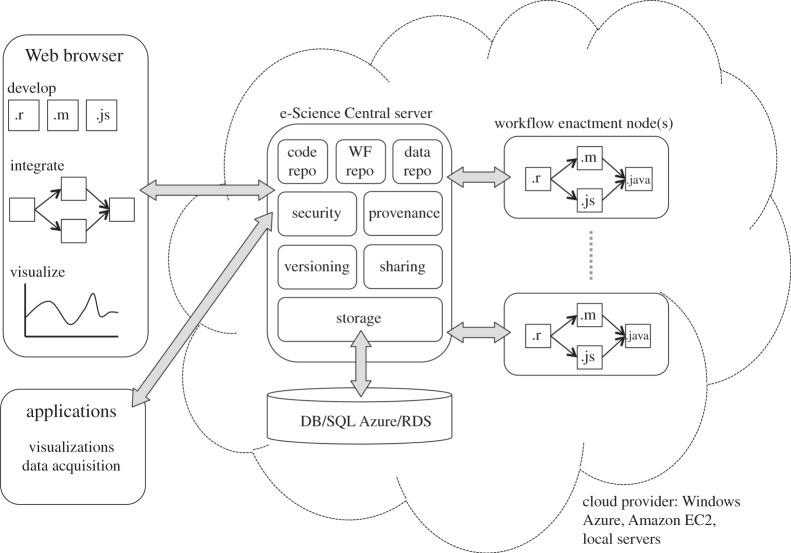
e-Science Central architecture.

The scientific platform offered by e-SC gives science application developers a high-level, cloud-based platform on which to build their applications. The platform architecture, including the main services within it, are shown in figure 1. These are built on processing and storage facilities provided by an underlying IaaS cloud (e.g. Amazon AWS or Windows Azure).

### (a) Storage and security

For intellectual property protection, all data are versioned and when a file is stored, if a previous version exists, then a new version is automatically created. This is important for allowing users to work with old versions of data, services and workflows, so supporting the reproduction of experiments, and investigations into the effects of changes to data and analysis processes over time.

In order to support users of e-SC in both industry and academia who require the ability to protect their data and analysis processes until they choose to publish, e-SC provides fine-grained security control. Security within e-SC is modelled around groups and user-to-user connections within a social network similar to that provided by myExperiment [7], which has demonstrated the potential of social networking to support collaboration in scientific research. These social networking connections are used as the basis for users to control the security of all information, including data, services and workflows. For example, scientists working together on a project could be assigned to a group and then data and analysis code could be shared exclusively between members of that group.

Data security has been implemented in the e-SC platform, rather than depending on the capabilities of the underlying storage system. This allows a range of underlying cloud storage services to be used, with no dependencies on the security mechanisms they natively offer. Drivers are currently available for the local file systems Amazon S3 and Azure Blob Store.

The default security for data is that it is private to the owner (i.e. the user who uploaded it), but at any time the owner can choose to share the data with others to whom they are connected, or to everyone in groups that they are members of or to make it public. Public data are reachable through Web search engines, so satisfying a common requirement of institutions and funding bodies that selected data should be openly available to encourage dissemination and support ‘open innovation’ [8].

All entities in the system (users, groups, data, workflows and services) are represented internally as subclasses of a single object. This allows them all to be guarded by a single security mechanism that checks every access. Access control lists (ACLs), associated with each object, are respected by this core object access code. These express security policies in terms of the actions that specific users and groups are allowed to perform on that resource. The actions can be: Read, Write, Delete and Add (allowing the addition of data to a container resource, e.g. a folder). The ACLs are stored in a database as triples comprising: the identity of the resource, the identity of the user or group and the action permitted. The architecture for this feature has been adopted from our earlier GOLD project [9].

All data can have associated metadata, which allows them to be described and discovered by the e-SC search engine. Two options are supported.

**Tagging** Users can add arbitrary tags to describe their data.
**Metadata documents** Users can upload metadata documents and associate these with data. This allows, for example, XML files to be used to describe data in accordance with a pre-defined schema. XPath [10] can then be used to perform structured searches for data.

As e-SC supports the storage, analysis and sharing of data, there is the opportunity to collect very rich provenance information. The e-SC provenance service records all system events such as data access, workflow execution and interactions through the social networking service. This is widely exploited in e-SC; for example, the Web interface allows the user to view the history and life cycle of every piece of data within the system, including who created it, who downloaded it, what version of which services (in what workflow) accessed the data and who the data have been shared with. This allows, for example, scientists to reproduce experiments, ascertain which files have been derived from a particular version of a service (perhaps now known to have a bug) and see who else has accessed files they have made available.

This provenance data model is based on the Open Provenance Model (OPM) v. 1.1 [11]. Data can be exported in the standard OPM format for analysis using tools such as the OPM toolbox, for example, to produce a directed acyclic graph of the history of an object.

### (b) Workflows

**Figure 2.**
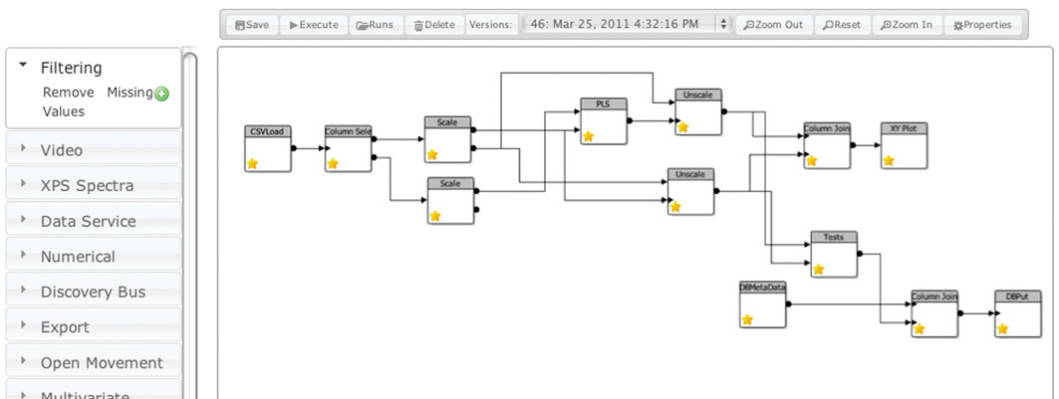
The e-Science Central workflow editor showing one of the chemical modelling workflows. (Online version in colour.)

A key feature of e-SC is that it allows users to process and manipulate data, rather than just to share them as is the case with many collaboration systems. Its in-browser workflow editor (figure 2) allows users to build a workflow by dragging services (either uploaded by themselves or shared by other users) from the structured list on the left of the screen, and connecting them together. The user can then execute the workflow, and the results are then displayed within the Web application and also stored within the e-SC file system for later use. This approach is well suited to situations where data can be processed automatically in batches without user interaction such as model building, data filtering and preprocessing and chart generation.

The workflow engine provides the means to scalably execute code in e-SC. It differs from engines such as Taverna [12] and Kepler [13] in that it has a browser-based editor, and automatically deploys services on multiple machines in the cloud as required to support its concurrent execution in multiple workflows. This concurrent execution differs from processing data using an approach such as MapReduce in that each processing node only requires the installation of a relatively small workflow engine service. Once the workflow engine service is configured any actual data-processing code required by workflows is downloaded and installed on demand as workflows execute. This avoids the need to configure all of the data-processing nodes with the software and tools required in advance.

Workflow services contain input and output ports, which can then be linked together. The input and output ports are able to restrict the types of data that can be sent to them, meaning that only compatible ports can be connected. Currently, the e-SC workflow engine supports the data types for ports shown below.

**data-wrapper** A rectangular grid of data. Data are typically arranged with each column representing a single attribute, with instances of the attributes arranged in rows. The data-wrapper type offers the option to stream large datasets through services in chunks of rows. CSV files are a typical example of this type of data.
**file-wrapper** A reference to a file, or list of files. The internal structure of the data is opaque to the workflow system; so no streaming is possible. Interpretation of the data is left entirely to the service code. An example of this type of data would be an image or text file.
**object-wrapper** An arbitrary, serialized Java object that can contain parameters or sets of parameters. These are usually used to pass complex objects (for example, entire models, or sets of scaling parameters) between services.

In the current implementation, all the workflow services within a single invocation of a workflow execute on the same cloud node, and so the intermediate data are passed between them via a temporary workflow invocation folder held on the filesystem of that node.

**Figure 3.**
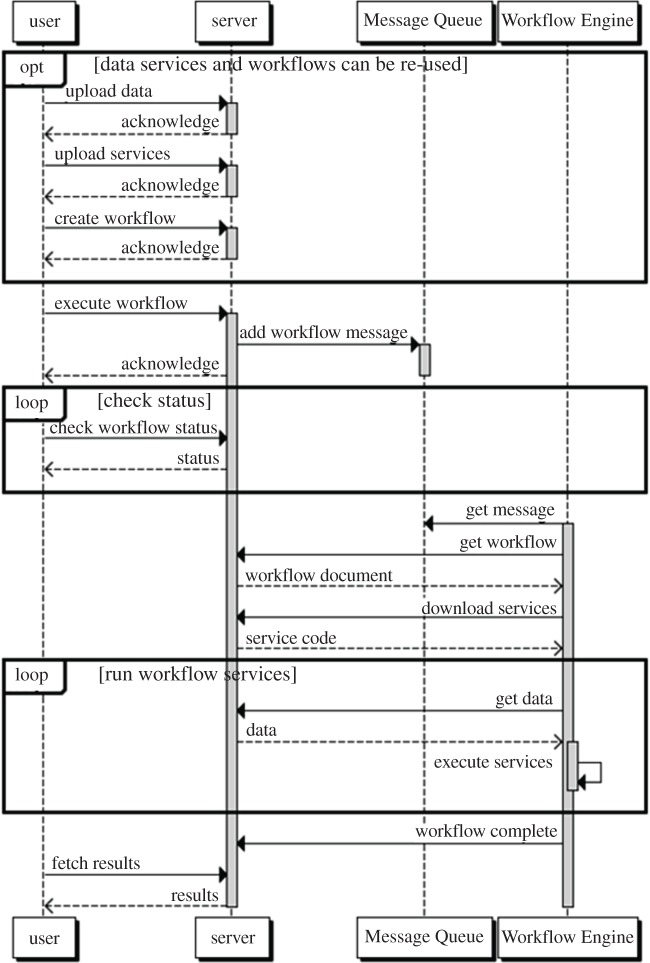
User and system interactions.

When a workflow is invoked, a series of operations takes place (figure 3). This sequence starts with a user uploading data to be analysed, uploading the workflow services required to operate on the data (if not already present) and defining a workflow to perform this analysis (if a suitable workflow does not already exist). A request to execute the workflow is then queued on a Java Message Queue and retrieved by one of the workflow engines. Once a workflow engine accepts this message, the workflow definition, data and services are downloaded, and the workflow is executed. Once the workflow is complete, the results are returned to the server and the user can retrieve them later.

One of the most common use cases for e-SC is to provide a data analysis back end to a standalone desktop or Web application. To support this pattern of use, the e-SC API provides a set of workflow control methods and data structures. The API provides a number of functions to support the execution of workflows and manage results. Using the API it is possible to upload new workflows and services, apply workflows to data files and dynamically configure workflow parameters prior to execution.

## 3. The e-Science Central application programming interface

While the platform services are made available to users through the Web application described in §1, programmatic access is also provided through a representational state transfer API to enable developers to build scalable, secure cloud applications. Security is managed by Java and .Net client libraries, which also give developers an object-oriented version of the API.

### (a) The application programming interface

Data passed to e-SC via the API is formatted as simple XML. The standard form for passing information is as lists of objects, embedded within the XML document. Each of the objects recognized by the e-SC API follows the same format, and there are object types for users, groups, folders, workflows, documents and document versions.

The API is implemented as a set of Java servlets [14] that interact directly with the application logic of e-SC via a set of Enterprise JavaBeans [14], which manipulate the underlying database (figure 1). These servlets are configured to intercept requests matching specific URL patterns. For example, there is a servlet configured at the base URL: /data/*, which provides access to documents held within the system. Using this pattern, version four of a document with the identifier f9f9e0005 is located at the URL: /data/f9f9e0005/4. The other servlets implementing the API intercept requests at similarly formatted URLs for users, workflows, groups, etc.

### (b) Application programming interface security

In order to make use of the e-SC API, the developer of the API client application is first required to register the application with e-SC. This registration process requires an application name and a URL at which the application is located. In addition, the permissions necessary for the application to operate must be specified. These permissions are used to express whether the application is, for example, permitted to access files from the system, execute workflows, etc. Once the application registration has been completed, an application identifier and key are made available. These are 32 character hexadecimal strings and are used to sign requests and data posted to the API servlets.

The data sent to the server are signed by calculating the MD5 hash [15] of the posted XML data plus the application identifier plus the URL information plus a sequence number. This signature, the application identifier and the sequence number are then added to the HTTP request data as header parameters. The server then validates this signature against the target URL, application identifier, sequence number and posted XML data. If the signature matches the value calculated by the server, then a check on the sequence number is made. The sequence number appended to the call data is used to prevent replay attacks on the server. For each registered application, the main database keeps a record of the last sequence number used by that application. For each request, this sequence number must increase in order for a request to be processed. The Java client library generates a sequence number derived from the system time.

## 4. Application programming interface case studies

The e-Sc API was designed to support three ways in which code developed by users could interact with e-SC.

— Standalone application: a standalone application is a separate website or desktop application that makes use of the e-SC data storage facility and/or workflow enactment engine.
— As an application appearing within the e-SC Web pages: e-SC allows code deployed on external Web servers to display information within the main website.
— As a block in a workflow: workflow blocks provide the main route by which users can incorporate code into e-SC. Typically, workflow blocks operate upon data supplied to them by other workflow blocks; however, blocks written in Java are provided with an API object that can be used to interact with the core of e-SC.

This section of the paper presents a case study for each of the API usage scenarios described earlier. Three case studies will be considered: the first is a medical data capture application that acts as a standalone system; the second is a spectral data visualization component that is hosted within the e-SC website; and the third is a chemical property modelling study that makes use of custom workflow blocks to execute multiple workflows in parallel on up to 200 CPUs.

### (a) Medical data capture application

Understanding patient activity levels is important to assessing key lifestyle variables linked to obesity, diabetes and cardiovascular disease. The MOVEeCloud project [16] makes use of wrist worn accelerometers that measure patient movement information over a period of weeks. These data are then extracted from the accelerometers and processed in order to analyse activity characteristics, including exercise and sleep. This processing is done via a set of e-SC workflows.

When a patient arrives at a clinic, the nurses and healthcare workers use a desktop application to extract the sample data from the measurement device and check that the data are of sufficiently high quality (i.e. that data are present for the entire sampling period). Once validated, the desktop application uploads these data using a C# version of the API client to e-SC. Upon successful upload, two workflows are automatically applied to these data: the first splits the data into one day sections and displays a subsampled set of data for each date on a separate graph, while the second processes the data in order to emulate an existing commercial accelerometer watch (the ActiWatch [17]). This processing step is required in order to compare data collected using more modern capture devices with that published in the literature collected using the older ActiWatch.

The dataset collected during the MOVEeCloud project is larger than that encountered during other e-SC applications to date. A typical data collection run with a single patient requires the watch to be worn for a two-week period. Data are sampled at 100 Hz on three axes, which yields a data file with approximately 120 million rows. The size of data files generated requires a different upload mechanism from the standard HTTP POST method used on the e-SC website. In order to accommodate these large data volumes, the desktop application uploads data using an HTTP stream attached directly to the back-end storage system (see figure 1). Processing files of this size also requires workflow blocks that are able to stream data in chunks through the code—this is supported by the workflow engine incorporated in e-SC, and the existing data processing code was modified to operate on a moving window of data as opposed to an entire file. Using this approach, the ActiWatch emulation workflow takes approximately 30 minutes to execute for each data file. This was deemed acceptable as the actual analysis of the collected data takes place some time after the raw data is uploaded and the turnaround time between collection and analysis is not critical to researchers.

The API methods used by this application are described in table 1.

**Table 1.** API functionality used in the medical data capture application.

API functionality	purpose
user authentication	allows the desktop application to authenticate the data gathering technicians
data upload	allows large data files to be uploaded to the system via a stream
workflow enactment	allows data processing workflows to be applied to newly uploaded data
data download	allows the generated graphs and ActiWatch emulated data to be downloaded and analysed externally

This application has been in use for six months as part of a pilot investigation, and a full rollout is the subject of a future project.

### (b) Spectral data visualization

The e-SC system has been adopted by the UK National X-ray Photoelectron Spectroscopy Centre based at Newcastle University [18]. Within this project, e-SC is being used as a central data repository and also as a means to provide specialized workflows to process collected data into one of the standard spectral data formats adopted by the project. Users of the X-ray photoelectron spectroscopy (XPS) facility manage their data using the standard file management tools provided by e-SC.

**Figure 4.**
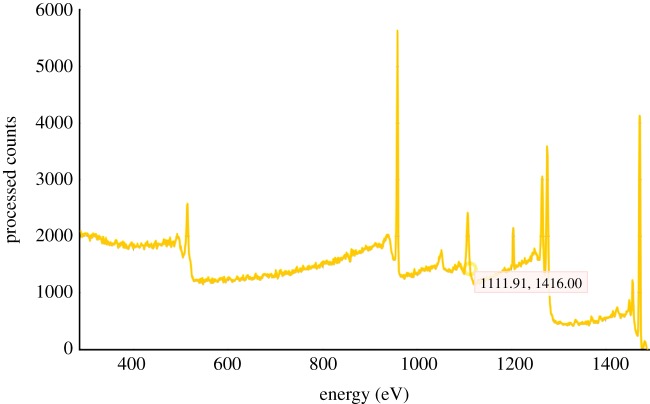
XPS spectral data viewer. (Online version in colour.)

Once data have been converted to the required ISO format [19], a visualization plugin has been provided that can be used within the e-SC data browser. The Web application providing this functionality is hosted on a dedicated e-SC server and has been registered with an e-SC system by providing a URL that accepts references to the data to be visualized. Whenever a visualization request is made to the application, a conversation takes place between the application and the e-SC server. This conversation creates an authenticated API connection that can retrieve the data to be plotted from the user's data folder and display them as a JavaScript chart presented within an iFrame in the e-SC file browser (figure 4).

The API features used by this application are described in table 2.

**Table 2.** API functionality used in the spectral data visualization application.

API functionality	purpose
API link authentication	provides an API connection for the visualization server that allows access to files owned by the user accessing the service
website integration	allows the visualization application to specify the MIME type of documents that it can process
data download	provides access to the file to be visualized

This deployment of e-SC will be maintained throughout the five year life of the XPS analysis centre at Newcastle, and its functionality will be expanded over time as more analysis and visualization tools are developed.

### (c) Chemical modelling

This subsection describes the use of e-SC to replace an existing scientific application that builds models of chemical properties. The aim was to speed up the application so that it could process the vast amounts of new chemical activity data that had recently been published in a reasonable time. The trigger for the work was an estimate that on its existing single-server architecture, the application would take five years to process the new data. The application uses quantitative structure–activity relationship (QSAR) [20] in order to identify new drugs that may block certain classes of cancer-causing kinase enzymes. QSAR correlates the chemical structure of compounds with properties such as reactivity or biological activity. For example, as the number of carbon atoms in a hydrocarbon increases, so does its boiling point. There are over 3000 descriptors that can be used to relate the quantifiable structural properties of a compound to its more complex properties such as biological activity. This, coupled with the wide range of different modelling algorithms, makes it computationally expensive to generate high-quality predictive models.

**Figure 5.**
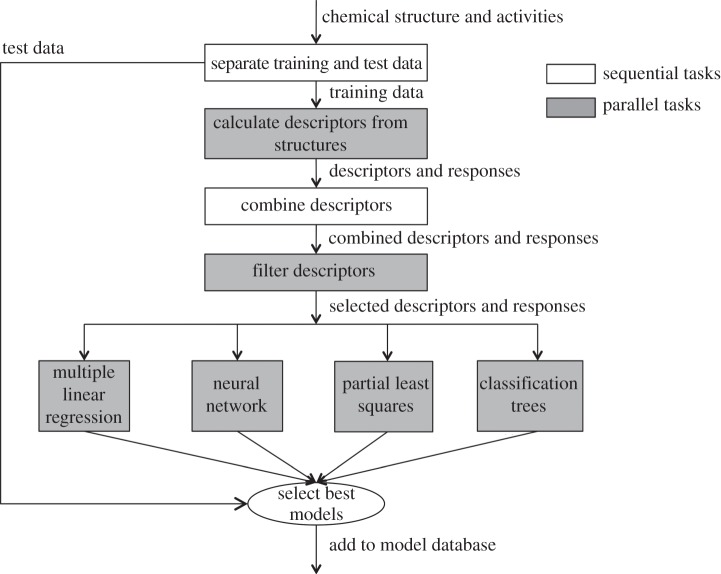
The Discovery Bus modelling process.

The chemists collaborating in this case study already have a system to automate the process of building QSAR models, which is referred to as the Discovery Bus [20]. At the highest level, the operation of the Discovery Bus can be represented using the flow chart shown in figure 5.

Because of the parallel nature of many of the most CPU-intensive aspects of the Discovery Bus model-building process (the shaded blocks in figure 5), there is the potential to exploit the cloud to speed up the model building. Initial work using the existing system running in an unmodified form on Amazon EC2 virtual machines did not demonstrate a satisfactory performance improvement (the limit of scalability was reached using only 20 workflow execution machines). In an attempt to improve this performance, the Discovery Bus workflow was re-implemented as a hierarchy of e-SC workflows.

At the top level of the modified system is a single workflow that operates on every available dataset. This workflow then initiates a new execution branch for each dataset that runs concurrently to calculate descriptors and, ultimately, to construct and validate a set of models (the workflow is shown in figure 2). The opportunities for parallelization in this system arise from the fact that each set of data can be treated independently. Thus, when the first workflow completes, there are several thousand independent workflow requests in the execution queue.

To create this system, a number of additional workflow blocks were developed. These blocks used the API provided to add new workflow execution requests to the workflow queue. At its peak, during processing 480 datasets, there were over 7000 workflow requests in the queue, each of which could potentially have been executed in parallel.

**Figure 6.**
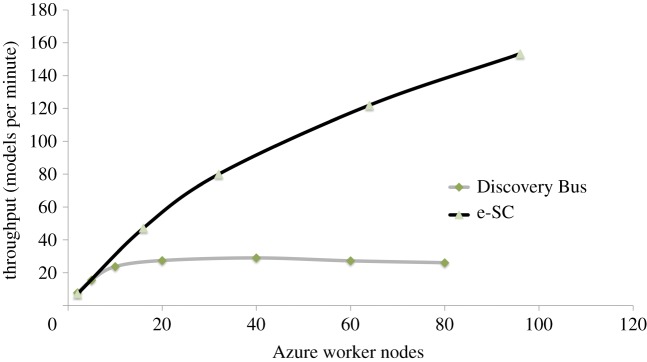
Scalability comparison between Discovery Bus application and e-SC. (Online version in colour.)

In order to investigate the scalability of this system, the e-SC platform was configured to run the workflows concurrently on up to 100 Windows Azure nodes (each containing two CPU cores), and figure 6 shows a comparison of the performance of the updated system with the original Discovery Bus application.

The API features used by this application are described in table 3.

**Table 3.** API functionality used by the chemical modelling application.

API functionality	purpose
data upload	allows a workflow to communicate with child workflows by uploading data to be analysed before the child workflow is executed
workflow execution	allows the top-level workflows to invoke multiple copies of modelling and descriptor calculation workflows in parallel

Our calculations show that the updated e-SC-based system could process the entire ChEMBL dataset [21] in approximately 14 h as opposed to the five years estimated for the original system. This modelling exercise will be performed whenever a new ChEMBL dataset is released: it is therefore an ideal cloud application as resources can be acquired, and paid for, only when new data become available.

## 5. Conclusions

This paper has described e-SC and its API in terms of both its science PaaS and its SaaS applications. The aim has been to make it easier for scientists to store, share and analyse their data, and for developers to create new scientific services and applications. We believe that the design and deployment of a science cloud platform, above the cloud infrastructure platforms that are now commercially available, has been important for achieving these goals. We also believe that by developing applications that run within e-SC, users can have some degree of independence in their choice of cloud providers, as there are versions of the software that can be hosted on Amazon EC2, Microsoft Windows Azure and local servers.

The case studies have demonstrated that the e-SC platform and API are suitable for the usage scenarios originally envisaged, and can be applied to a range of scientific applications. In particular, the chemical modelling case study presented has demonstrated the potential of the system to scale to a significant number of computational resources.
